# Melanosomes as Antigen‐Presenting Decoys in Melanoma Immune Evasion

**DOI:** 10.1002/mco2.70797

**Published:** 2026-07-06

**Authors:** Yingqi Kuang, Fangfang Zhou

**Affiliations:** ^1^ Department of Dermatology, The First Affiliated Hospital of Soochow University Institutes of Biology and Medical Science Suzhou Medical College, Soochow University Suzhou China; ^2^ Biomedical Basic Research Center (BBRC) of Jiangsu Province Suzhou China

1

In a recent study published in *Cell*, Chemla et al. uncovered a previously unrecognized immune evasion mechanism whereby melanoma exploits secreted melanosomes to hijack antigen‐specific CD8^+^ T cells [1]. This interaction impairs tumor antigen recognition, reduces T‐cell activation, and increases apoptosis, ultimately promoting immune escape. These findings suggest that targeting melanosomes‐mediated interactions may enhance the efficacy of melanoma immunotherapy (Figure 1).

**FIGURE 1:**
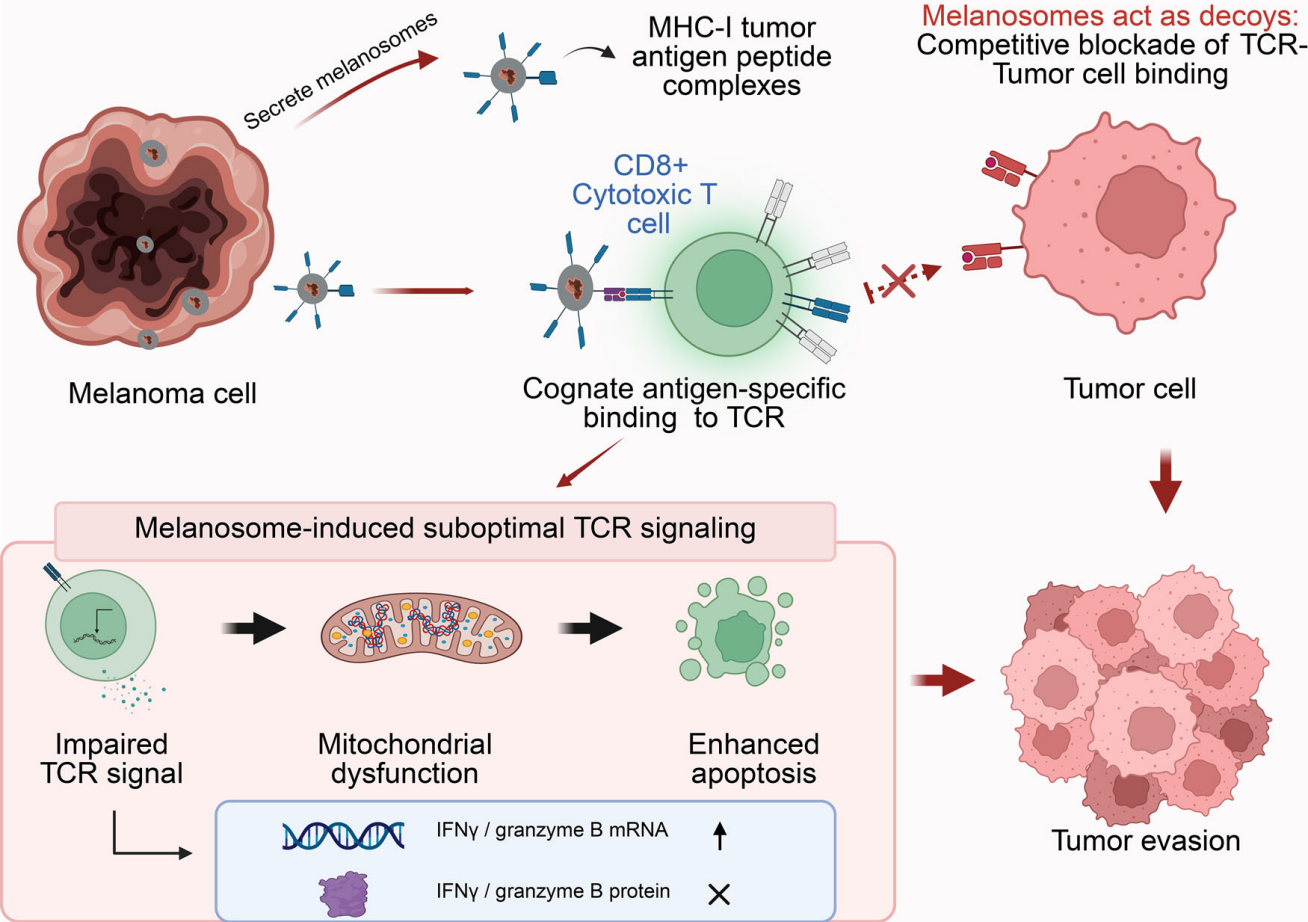
Melanosomes facilitate melanoma immune evasion through competitive TCR inhibition. Melanoma cells secrete melanosomes carrying MHC‐I–tumor antigen complexes that specifically bind to T‐cell receptors (TCRs) on CD8^+^ cytotoxic T cells. These melanosomes act as competitive decoys that physically occupy TCRs (occupied TCRs shown in the figure), thereby blocking the direct recognition of tumor cells (indicated by the red cross, **×**). This interaction induces suboptimal TCR signaling and mitochondrial dysfunction, ultimately leading to enhanced T‐cell apoptosis. While effector transcription is active (up arrow, ↑, for mRNA levels), the translation of IFN‐γ and granzyme B proteins is severely impaired (black cross, ✕), promoting melanoma immune evasion and tumor progression. Created with BioRender.com.

Melanoma is a highly aggressive tumor caused by the malignant transformation of melanocytes. It is also one of the deadliest skin cancers with an average annual growth rate of 4%–6% in incidence [2]. Although immunotherapy has developed rapidly, nearly half of patients still do not respond to the treatment [3]. The mechanisms underlying immune evasion that drive this resistance remain incompletely understood.

As a malignancy of melanocytic origin, melanoma is distinguished by the presence of melanosomes—lineage‐restricted, pigment‐producing organelles essential for melanin synthesis and storage. Beyond their canonical intracellular role, melanoma cells actively secrete melanosomes into the tumor microenvironment. Accumulating evidence has demonstrated that melanoma‐derived melanosomes drive fibroblast transformation, promote lymphomagenesis, induce macrophage diversification, and confer therapeutic resistance, further contributing to melanoma drug resistance by exporting chemotherapeutic agents [4, 5, 6]. However, despite these well‐established protumorigenic functions, whether melanoma‐derived melanosomes directly regulate antigen‐specific CD8^+^ T‐cell function and antitumor immunity has remained unclear.

Previous studies showed that melanosomes are secreted near melanoma cells and their expression correlates with poor prognosis in melanoma patients. In the Cancer Genome Atlas datasets, high expression of melanosome‐associated genes is significantly correlated with poor prognosis. Non‐responders to immunotherapy showed increased “melanosome‐loaded T cells,” which were smaller and exhibited features of T‐cell exhaustion. To verify that the melanosomes can directly interact with CD8^+^ T cells, the authors isolated the melanosomes from patients and directly observed their interaction with Tumor‐infiltraing lymphcttes (TILs). Imaging and Fluorescence‐Activated Cell Sortin (FACS) secretion analysis indicated that the melanosomes directly bound to CD8^+^ T cells, and the binding efficiency was significantly higher than that of CD8^+^ T cells binding to tumor cells themselves.

To clarify the role of melanosomes in tumor immune evasion, the authors inhibited melanosomes secretion by targeting tyrosinase with kojic acid or using CRISPR‐Cas9. This intervention significantly inhibited tumor growth and prolonged the survival of mice, but it had no obvious effect on the MC38 colorectal cancer model. The antitumor effect was dependent on CD8^+^ T cells, indicating that it was not the melanoma itself that played a role, but the melanosomes secreted by the melanoma that exerted the effect. Moreover, melanosomes inhibition could increase the proportion of NK cells and promote the shift of CD4^+^ T cells toward the Th1 phenotype, further supporting the crucial role of melanosomes in the immune escape of melanoma.

Based on this, the researchers further explored the direct interaction mechanism between melanosomes and T cells, isolated melanosomes and exosomes secreted by human (MNT1) and mouse (B16F10) melanoma cells, and conducted proteomic analysis on them. Interestingly, both melanosomes were significantly enriched with various immune‐related proteins compared to exosomes, especially MHC‐I molecules closely related to antigen processing and presentation. Further analysis revealed that this characteristic was only present in melanosomes derived from melanoma cells and was not observed in melanosomes derived from normal melanocytes. Further experiments found that the MHC‐I on the surface of melanosomes had successfully loaded antigen peptides and possessed the structural and functional basis for specific binding with the TCR of CD8^+^ T cells. Expanding to the endogenous system, the significant co‐localization of melanosomal MHC‐I and TCR clarified that they directly interacted through the “MHC‐antigen peptide‐TCR” pathway.

Authors found the MHC‐I binding peptides on the surface of melanosomes showed a high degree of consistency in characteristics such as length distribution, hydrophobicity, and retention time with the MHC‐I peptide spectrum on the surface of tumor cells. Although there are different distributions of MHC I ligand spectra between melanoma cells and melanosomes, a large number of peptide chains from melanosomes sources overlap with those of the parent cells, indicating that they originate from the entire cell ligands. At the same time, multiple mutated‐derived neoantigens were also discovered, some of which are shared with tumor cells. These results indicate that melanosomes not only possess complete antigen presentation capabilities but also accumulate highly immunogenic antigens, providing a key material basis for their specific attraction and “capturing” of antigen‐specific CD8^+^ T cells.

Additionally, the study showed that melanosomes block anti‐tumor immune responses by occupying the TCR binding sites. This prevents effective immune synapse formation between T cells and tumor cells, resulting in immune evasion. The combination of melanosomes and T cells led to reduced TCR signaling, decreased mitochondrial activity, and increased apoptosis.

This study reveals a novel mechanism whereby melanoma‐derived melanosomes act as “antigen‐presenting decoys” to directly inhibit tumor‐reactive CD8^+^ T cells. Unlike previous studies that mainly focused on indirect regulation through stromal cells—such as fibroblasts and macrophages, affecting polarization or therapy resistance via miRNAs, protein kinases, or other cargo—this work demonstrates that melanosomes can directly misdirect antigen recognition, impair TCR signaling, and reduce mitochondrial function, thereby promoting immune escape. This highlights a new, direct layer of immune regulation at the antigen presentation and TCR recognition stage, expanding the functional scope of melanosomes beyond microenvironment modulation.

The innovations of this study are clear. First, it establishes a direct inhibitory mechanism on CD8^+^ T cells, complementing prior knowledge of stromal‐mediated immune regulation. Second, it identifies the site of action at the early stage of antigen recognition, providing mechanistic insight into the initiation of tumor immune escape. Third, it suggests potential interventions targeting melanosomes secretion, surface antigen‐presenting molecules, or melanosomes–T cells interactions to enhance immunotherapy efficacy.

Nevertheless, limitations remain. Tumor‐specific targeting is crucial because normal melanocytes also secrete melanosomes, raising concerns about off‐target effects. The in vivo delivery, clearance, and tissue distribution of melanosomes require further study. The universality of this mechanism across melanoma subtypes and progression stages, the origin of MHC‐I molecules on melanosomes, and the downstream signaling pathways leading to T cell dysfunction remain unresolved.

From a translational perspective, melanosome‐mediated T‐cell inhibition may represent a novel subtype of immunotherapy resistance, distinct from classic mechanisms such as MHC‐I deficiency or checkpoint‐mediated T‐cell exhaustion. Targeting melanosomes could be combined with existing therapies: immune checkpoint inhibitors to relieve T‐cell exhaustion while reducing antigen misdirection, or adoptive T‐cell therapy to enhance persistence and efficacy. Patient stratification based on melanosomes load or antigen presentation characteristics may help identify those most likely to benefit from such combined strategies.

Overall, this work integrates mechanistic insight with translational relevance, establishing a foundation for future research on tumor‐derived organelles as mediators of immune escape and guiding the development of targeted immunotherapy strategies.

## Author Contributions

Y.K. wrote the manuscript and prepared the figure. F.Z. provided valuable discussion. Both authors have read and approved the final manuscript.

## Funding

This work was supported by a special program from the Ministry of Science and Technology of the People's Republic of China (2022YFA1105200), the Natural Science Foundation of Jiangsu Province (No. BK20255001), and the Priority Academic Program Development of Jiangsu Higher Education Institutions.

## Ethics Statement

The authors have nothing to report.

## Conflicts of Interest

The authors declare no conflicts of interest.

## Data Availability

The authors have nothing to report.
